# Clinician care priorities and practices in the fourth trimester: perspective from a California survey

**DOI:** 10.1186/s12884-024-06705-7

**Published:** 2024-07-25

**Authors:** Sylvia Guendelman, Serena Xinzi Wang, Maureen Lahiff, Lawrence Lurvey, Hayley E. Miller

**Affiliations:** 1 School of Public Health, University of California, Berkeley, 2121 Berkeley Way, Room, 6124, Berkeley, Ca 94720-7360 USA; 2 School of Public Health, University of California, Berkeley Class of 2025, 2121 Berkeley Way West, Berkeley, Ca 94720-7360 USA; 3 School of Public Health, University of California, 2121 Berkeley Way, Room 5302, Berkeley, Ca 94720-7360 USA; 4 Kaiser Permanente West Los Angeles, 6041 Cadillac Ave, Los Angeles, Ca 90034 USA; 5 Center for Academic Medicine, Stanford University School of Medicine, 453 Quarry Road, Palo Alto, Ca 94305-5317 USA

**Keywords:** Postpartum, Clinician care priorities, Care practices, Clinical care guidelines, California, OB/GYNS, Midwives

## Abstract

**Background:**

Professional societies such as the American College of Obstetricians and Gynecologists (ACOG) promote the idea that postpartum care is an ongoing process where there is adequate opportunity to provide services and support. Nonetheless, in practice, the guidelines ask clinicians to perform more clinical responsibilities than they might be able to do with limited time and resources.

**Methods:**

We conducted an online survey among practicing obstetric clinicians (obstetrician/gynecologists (OB/GYNs), midwives, and family medicine doctors) in California about their priorities and care practices for the first postpartum visit and explored how they prioritize multiple clinical responsibilities within existing time and resources. Between September 2023 and February 2024, 174 out of 229 eligible participants completed the survey, a 76% response rate. From a list of care components, we used descriptive statistics to identify those that were highly prioritized by most clinicians and those that were considered a priority by very few and examined the alignment between prioritized components and recommended care practices.

**Results:**

Clinicians were highly invested in the care components that they rated as most important, indicating that they always check these components or assess them when they perceive patient need. *Depression and anxiety*,* breast health/breast feeding issues*,* vaginal birth complications and family planning counseling* were highly ranked components by all clinicians. In contrast, clinicians more often did not assess those care components that infrequently ranked highly among the priority listing, consisting mainly of social drivers of health such as screening and counseling for *intimate partner violence*,* working conditions and food/housing insecurity*. In both instances, we found little discordance between priorities and care practices. However, OB/GYNs and midwives differed in some care components that they prioritized highly.

**Conclusions:**

While there is growing understanding of how important professional society recommendations are for maternal-infant health, clinicians face barriers completing all recommendations, especially those components related to social drivers of health. However, what the clinicians do prioritize highly, they are likely to perform. Now that Medi-Cal (Medicaid) insurance is available in California for up to 12 months postpartum, there is a need to understand what care clinicians provide and what gaps remain.

**Supplementary Information:**

The online version contains supplementary material available at 10.1186/s12884-024-06705-7.

## Introduction

There is a growing understanding of the importance of postpartum care beyond the traditional six-week single visit. Evidence shows that common postpartum health challenges such as mood disorders, medical co-morbidities, lactation difficulties, fatigue, pain, and incontinence affect almost three-quarters of all birthing people [1]. Approximately, one-third of patients continue to experience childbirth-related complications after the standard single 6-week visit and require emergency department services [2]. The National Committee for Quality Assurance (NCQA) recently set three standards for postpartum care: the timeframe for a single postpartum visit between 7 and 84 days after delivery, depression screening and follow-up, and contraceptive counseling [3]. These standards constitute performance measures frequently tied to financial incentives for insurers, health plans and clinicians [2].

In 2018, the American College of Obstetricians and Gynecologists (ACOG) proposed the Fourth Trimester as the paradigm for postpartum care. ACOG recommends that all birthing people have an initial assessment within 21 days after birth, followed by ongoing care as needed and a comprehensive assessment by the start of the 12th week after birth. In addition to mood and emotional well-being, contraceptive care and birth spacing, a comprehensive visit should address infant care and feeding, sleep and fatigue, physical recovery from birth, chronic disease management and overall health management [4].

California prioritized postpartum care, extending Medicaid postpartum coverage in 2022 from 60 days to 12 months [5, 6]. Under this new policy, birthing people with Medi-Cal insurance (Medicaid’s program in California) can receive more than a single postpartum clinical visit at 6 weeks —the traditional norm demonstrated to be insufficient— [7, 8] and expanded health insurance up to 12 months postpartum [6].

Clinician compliance with these guidelines and care coverage opportunities remains unknown, despite increasing rates of maternal mortality and morbidity, large racial inequities in maternal health outcomes, and growing behavioral health problems [9, 10]. Clinicians are often burdened by competing economic and time demands to fulfill the numerous recommendations. Workforce shortages, worsened by the Covid-19 pandemic, may exacerbate the pressure. In a US survey administered in 2018, clinicians reported that they nearly always addressed pregnancy and birth complications and screened for depression and contraceptive care at the 6-week postpartum visit [11]. Of the 17 elements clinicians were asked to prioritize, none were rated as having no or low importance, despite the multiple demands on their time and resources, possibly indicating overreporting of socially desirable behaviors. However, clinicians reported that tradeoffs do occur as there was discordance between some care practices and those considered high priority by the clinician or recommended by ACOG [11].

In this article, we examine the postpartum care priorities and care practices performed by practicing obstetric care clinicians in California and explore how they juggle multiple priorities within existing time and resources. Using a contemporary cohort of clinicians (mainly obstetrician gynecologists (OB/GYNs), midwives and family medicine doctors) we explore the following key questions regarding the first outpatient postpartum visit:

1. Among the many priorities, which rise to the top and which stay at the bottom?
2. Among the most frequently (and infrequently) prioritized components, at what rate is care performed?
3. Do care priorities and practices align or are there discrepancies between priorities and practices that suggest trade-offs due to competing multiple demands?
4. To what extent do priorities and care practices differ between OB/GYNs and midwives?

## Materials and methods

This cross-sectional, near-time study draws on data from the California Postpartum Care Survey, an anonymous, online survey of current clinical practices in the postpartum, perceived care priorities and gaps and opportunities to improve care. We developed survey content from previously published surveys, [11, 12] professional organizations [3, 4, 9, 10] and from three focus groups with seven practicing physicians who provided input on these issues during the formative stage of this project. The 32-question Qualtrics survey (Supplementary 1) was developed for the purpose of this study and took, on average, 12 min to complete and was active between September 1, 2023, and February 2, 2024.

### Recruitment and eligibility

We enlisted various leaders of professional organizations (ACOG District IX, California Maternal Health Quality Control Collaborative (CMQCC), March of Dimes (MOD), the California Department of Public Health (CDPH), the Preconception Health Council of California and Perinatal Services, and the UC Berkeley School of Public Health) to help us recruit eligible participants from their networks and promote the survey. Champions personally communicated with their contacts and sent out written announcements that provided a description and a link to the survey. Champions also encouraged their contacts to recruit other potential participants who met the eligibility criteria. Furthermore, we advertised our survey in Rounds, the monthly ACOG newsletter.

To be eligible for the study, participants had to be active clinicians providing obstetric care, including postpartum care in California. Participants gave informed consent when they initiated the survey and those who completed the survey became eligible for one of five drawings of $100 gift cards at the end of the data collection period. This study was approved by the Institutional Review Boards of the University of California, Berkeley (Protocol No. 2023-01-15992) and Stanford University (Protocol No. 71687).

Of the 353 potential respondents who opened the link to the survey, 124 were excluded from analysis because they did not meet the eligibility criteria, or they opted out of the survey and the eligibility criteria were unknown. An additional 55 participants were eligible but did not complete the survey. Based on the eligible responses, the overall response rate was 174/229 = 76%. Survey respondents did not differ from eligible non-respondents by clinician type. Clinicians were initially grouped into three study groups: OB/GYNs (*n* = 97), midwives (*n* = 61) and family medicine doctors (*n* = 12). An “All” group was created that included the three study groups plus four nurse practitioners.

### Survey measures

Drawing from a list of 26 care components, respondents were asked “*What are the top 5 postpartum topics that you prioritize reviewing with your patient during the first postpartum visit?”* From this question, we created two outcome measures: (1) the top five prioritized care components defined as those that at least 40% of respondents consider as highest priority; (2) Those care components that very few respondents (≤ 10%) consider a top priority.

A third outcome measure, the rate of practice of care components, was obtained from the question “*Do you check for the following elements at the first postpartum visit: Always*,* Only if the patient needs it*,* No but I delegate/refer or do not check”. C*omponents were selected from ACOG postpartum care practice recommendations [4, 9, 10] and included: Clinical elements *(c-section birth complications*,* vaginal birth complications*,* pregnancy-related complications*,* physical recovery after labor*,* physical/pelvic exam*,* remote blood pressure monitoring*,* chronic health conditions);* Behavioral (*depression and anxiety*,* substance use*,* smoking*,* maternal sleep*,* diet and weight trajectory*); Family planning *(counseling*,* contraceptive provision*,* resume sexual activity);* Infant health *(breast health/feeding*,* safe sleep*,* infant bonding);* Social *(social and emotional support*,* intimate partner violence*,* safe work environment*,* work evaluation*,* adverse childhood experiences (ACEs) evaluation*,* food and housing insecurity);* and Future care *(review birth experience and prepare for future pregnancies*,* development/implementation of a postpartum care plan that details patient’s needs*,* and transitioning to primary care).*

The key exposure examined in this article was the type of clinician. While all obstetric care clinicians provide medical care, support and guidance during the prenatal, labor and delivery and/or the postpartum period, midwives tend to care for low to moderate-risk patients. OB/GYNs are trained to additionally care for high-risk patients, perform surgeries such as cesarean sections and to manage pregnancy complications with medical interventions such as inductions and assisted deliveries [13]. Additional variables examined were the timing of the provision of the first postpartum care visit, the duration of that visit (in minutes), the type of practice setting, the practice location, and the proportion of patients in the practice with and without Medi-Cal coverage (Table 1). We refer to these domains as the “practice characteristics” or “practice environment”.

### Statistical analysis

We used descriptive statistics (mean and standard deviation for continuous variables, count and percent for categorical variables and median interquartile range for time in practice due to its skewed distribution) to summarize responses for all survey respondents and by clinician type. We compared differences between OB/GYNs and midwives performing Fisher exact tests or t-tests. We did not compare family medicine doctors with these two study groups because the small sample size yielded insufficient power. To examine whether the proportion of care practices differed between clinicians who rated a component as a top priority and those who did not, we created contingency tables that compared these two groups and followed up with Fisher exact tests. These comparisons allowed us to assess discrepancies in care priorities and actual practice. In addition, we explored the care practices associated with several components that very few clinicians (≤ 5%) considered to be highest priority for the first visit, despite their clinical relevance based on ACOG’s guidance [4, 9, 10]. Lastly, we qualitatively compared the highest priorities observed by clinician type to those observed according to practice setting, practice location, timing of the first postpartum visit and proportion of Medi-Cal patients served. These comparisons allowed us to identify the extent to which the scope of practice and the practice environment of midwives and OB/GYNs align with their care priorities.

**Table 1:** Clinician and practice characteristics

Sample Characteristics	All ^†^		OB/GYNs		Midwives		Family medicine doctors		*p*-value * (OB/GYNs vs. Midwives)
	*n*	%	*n*	%	*n*	%	*n*	%	
**Overall**	174	100	97	55.7	61	35.1	12	6.9	
**Age (in years) [mean(SD)]**	46.30(11.05)		45.62(11.11)		47.47(11.32)		43.67(10.29)		N.S. ^t^
**Self-identified gender**									N.S. ^f^
Woman	159	91.4	88	90.7	59	96.7	8	66.7	
Man	12	6.9	8	8.2	1	1.6	3	25	
Missing	3	1.7	1	1	1	1.6	1	8.3	
**Race/Ethnicity**									< 0.01 ^f^
Asian	18	10.3	13	13.4	2	3.3	2	16.7	< 0.05 ^f^
Black	9	5.2	9	9.3	0	0	0	0	< 0.05 ^f^
Hispanic	12	6.9	6	6.2	3	4.9	2	16.7	N.S. ^f^
Multiracial/Other	13	7.5	4	4.1	8	13.1	1	8.3	N.S. ^f^
White non-Hispanic	116	66.7	61	62.9	47	77	6	50	N.S. ^f^
Missing	6	3.4	4	4.1	1	1.6	1	8.3	
**Years practicing [mean(SD)]**	14.13(10.51)		14.40(11.02)		13.81(10.05)		11.09(9.47)		N.S. ^t^
**Practice setting**									< 0.0001 ^f^
University/Academic	50	28.7	43	44.3	5	8.2	0	0	< 0.0001 ^f^
Community hospital/ health center/clinic	41	23.6	16	16.5	16	26.2	9	75	N.S. ^f^
Staff model HMO	25	14.4	16	16.5	7	11.5	1	8.3	N.S. ^f^
Private practice	39	22.4	15	15.5	24	39.3	0	0	< 0.01 ^f^
Multi-affiliated/Other	18	10.3	6	6.2	9	14.8	2	16.7	N.S. ^f^
**Practice location**									< 0.0001 ^f^
Urban, large city	87	50	57	58.8	23	37.7	3	25	< 0.05 ^f^
Suburb, near large city	58	33.3	36	37.1	17	27.9	5	41.7	N.S. ^f^
Small city or town	28	16.1	3	3.1	21	34.4	4	33.3	< 0.0001 ^f^
Rural	0	0	0	0	0	0	0	0	
**Percentage of patients with Medi-Cal insurance**									N.S. ^f^
≤ 50%	98	56.3	61	62.9	34	55.7	1	8.3	
> 50%	69	39.7	32	33	24	39.3	11	91.7	
**First visit length [median(IQR)]**	20.00(10.00)		20.00(10.00)		60.00(55.00)		20.00(10.00)		< 0.0001 ^k^
**First visit timeframe**									< 0.0001 ^f^
Within 2 weeks	71	40.8	21	21.6	42	68.9	8	66.7	< 0.0001 ^f^
Within 3–5 weeks	36	20.7	25	25.8	8	13.1	2	16.7	N.S. ^f^
6 weeks − 12 weeks	26	14.9	18	18.6	5	8.2	1	8.3	N.S. ^f^
Depends on patient’s condition	41	23.6	33	34	6	9.8	1	8.3	< 0.01 ^f^
**Only 1 postpartum visit for low-risk patients**	57	32.8	45	46.4	10	16.4	0	0	< 0.01 ^f^
**Only 1 postpartum visit for high-risk patients**	18	10.3	11	11.3	6	9.8	1	8.3	N.S. ^f^

*N.S. indicates a non-significant result (p-value > 0.05) from t-test (^t^), Fisher’s exact test (^f^), or Kruskal Wallis test (^k^)

^†^All clinicians include OB/GYNs, Midwives, Family medicine doctors, and Nurse practitioners

**Only one OB/GYN did not respond for practice setting and practice location

## Results

Respondents were on average 46 years old and had 14 years of practice (Table 1). The majority (91.4%) self-identified as women, about two thirds were non-Hispanic White. The majority practiced in either academic settings (28.7%), community hospitals or clinics (23.6%) or private practices (22.4%). Half practiced in urban locations and only 16.1% in small cities or towns. Almost 40% had practices where > 50% of the patients were insured by Medi-Cal.

The duration of first postpartum visits were skewed so we report the median, which was 20 min. Notably, one third of clinicians offered their low-risk patients only one visit; 40.8% of the clinicians saw their patients within 2 weeks, 20.7% saw patients within 3 to 5 weeks and another 23.6% saw them according to the patient’s condition. Only 14.9% saw their patients for their first visit at 6–12 weeks (Table 1). Compared to midwives, OB/GYNs were more likely to be Black (*p* < 0.05) or Asian (*p* < 0.05), practice in academic settings (*p* < 0.0001), and in urban locations (*p* < 0.05). The median length of the first visit for OB/GYNs was 20 min vs. 60 min for midwives (*p* < 0.0001) and the timing of the visit for midwives tended to be earlier (*p* < 0.0001). More OB/GYNs reported that their patients received only one postpartum visit (46.4% versus 16.4% *p* < 0.01).

### Care priorities and care practices

The top priorities for the first postpartum visit among all clinicians were *depression and anxiety*,* breast health/feeding issues*,* vaginal birth complications*,* c-section birth complications*,* pregnancy-related complications*,* family planning counseling*,* social and emotional support*,* and physical recovery after labor (*Table 2). Out of these eight components, both OB/GYNs and midwives ranked *depression and anxiety*,* breast health/feeding issues*,* vaginal birth complications* and *family planning counseling* at the top. While OB/GYNs who see high-risk patients ranked *c-section birth complications* and *pregnancy-related complications* highly, only midwives ranked *social and emotional support* and *physical recovery after labor* in the top tier. Midwives additionally, ranked *maternal sleep* among the top priorities.

**Table 2:** Top priorities for all clinicians, OB/GYNs and midwives

Clinician Priority	All^†^ (*N* = 174)		Clinician Priority	OB/GYNs (*N* = 97)			Clinician Priority	Midwives (*N* = 61)	
	*n*	%		*n*		%		*n*	%
**Depression and anxiety**	**146**	**83.9**	**Depression and anxiety**	**86**	**88.7**		**Breast health, feeding issues**	**56**	**91.8**
**Breast health, feeding issues**	**109**	**62.6**	**Pregnancy-related complications**	**65**	**67**		**Depression and anxiety**	**45**	**73.8**
**Vaginal birth complications**	**91**	**52.3**	**C-section birth complications**	**63**	**64.9**		**Physical recovery after labor**	**40**	**65.6**
**C-section birth complications**	**87**	**50**	**Vaginal birth complications**	**59**	**60.8**		**Social and emotional support**	**34**	**55.7**
**Pregnancy-related complications**	**80**	**46**	**Family planning counsel**	**47**	**48.5**		**Maternal sleep**	**27**	**44.3**
**Family planning counseling**	**77**	**44.3**	**Breast health**,** feeding issues**	**40**	**41.2**		**Vaginal birth complications**	**25**	**41**
**Social and emotional support**	**73**	**42**	Contraceptive provision	35	36.1		**Family planning counsel**	**25**	**41**
**Physical recovery after labor**	**70**	**40.2**	Social and emotional support	32	33		C-section birth complications	15	24.6
Contraceptive provision	45	25.9	Physical recovery after labor	23	23.7		Review birth experience and prepare for future pregnancies	14	23
Maternal sleep	40	23	Chronic health conditions	21	21.6		Exercise and nutrition	13	21.3
Review birth experience and prepare for future pregnancies	27	15.5	Maternal sleep	11	11.3		Development/ implementation of a postpartum care plan	11	18
Chronic health conditions	25	14.4	Review birth experience and prepare for future pregnancies	11	11.3		Pregnancy-related complications	10	16.4
Development/ implementation of a postpartum care plan	21	12.1	*Development/* *implementation of a postpartum care plan*	*9*	*9.3*		Infant safe sleep	9	14.8
*Exercise and nutrition*	*16*	*9.2*	*Resuming sexual activity*	*5*	*5.2*		Infant bonding	9	14.8
*Resuming sexual activity*	*12*	*6.9*	*Intimate partner violence*	*5*	*5.2*		*Resuming sexual activity*	5	8.2
*Infant safe sleep*	*11*	*6.3*	*Substance use*	*5*	*5.2*		*Transitioning to primary care*	*5*	*8.2*
*Infant bonding*	*10*	*5.7*	*Transitioning to primary care*	*4*	*4.1*		*Contraceptive provision*	*4*	*6.6*
*Transitioning to primary care*	*10*	*5.7*	*Exercise and nutrition*	*3*	*3.1*		*Chronic health conditions*	*4*	*6.6*
*Intimate partner violence*	*8*	*4.6*	*Diet and weight trajectory*	*2*	*2.1*		*Intimate partner violence*	*3*	*4.9*
*Food and/or housing insecurity*	*5*	*2.9*	*Evaluate work environment*	*2*	*2.1*		*Food and/or housing insecurity*	*3*	*4.9*
*Diet and weight trajectory*	*5*	*2.9*	*Safe work environment*	*2*	*2.1*		*Diet and weight trajectory*	*2*	*3.3*
*Substance use*	*5*	*2.9*	*Infant safe sleep*	*1*	*1*		*ACE evaluation*	*1*	*1.6*
*ACE evaluation*	*2*	*1.1*	*Infant bonding*	*1*	*1*		*Substance use*	*0*	*0*
*Evaluate work environment*	*2*	*1.1*	*Food and/or housing insecurity*	*1*	*1*		*Evaluate work environment*	*0*	*0*
*Safe work environment*	*2*	*1.1*	*ACE evaluation*	*1*	*1*		*Safe work environment*	*0*	*0*
*Smoking*	*1*	*0.6*	*Smoking*	*1*	*1*		*Smoking*	*0*	*0*

*Top tier – ranked a top priority by at least 40% of respondents and shown in bolded font

**Bottom tier – ranked a top priority by less than or equal to 10% of respondents and shown in italicized font

^†^All clinicians include OB/GYNs, Midwives, Family medicine doctors, and Nurse practitioners

Various care components that were infrequently ranked as top priorities were *exercise and nutrition*,* weight and diet trajectory*,* resuming sexual activity*,* infant safe sleep*,* infant bonding*,* transitioning to primary care*,* intimate partner violence*,* food and housing insecurity*,* substance use*,* adverse childhood experiences (ACE evaluation)*,* work environment and smoking*. Both OB/GYNs and midwives similarly placed these components in the lowest tier with some exceptions: *exercise and nutrition*,* and infant bonding/sleep* were ranked higher by midwives. In addition, while midwives placed management of chronic disease in the lowest tier more OB/GYNs placed development of a postpartum care plan in the lowest tier (Table 2).

**Fig. 1:**
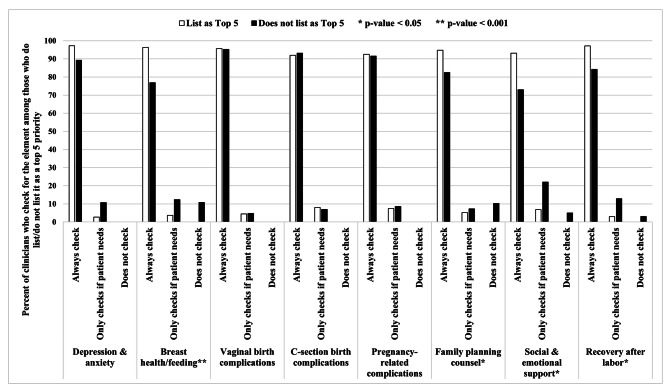
Clinician care practices among priorities rated as “top priorities” by ≥ 40% of all clinicians

Figure 1 shows that clinicians were actualizing clinical practices that were ranked among the top priority care components. Regardless of whether each component was ranked at the top, clinicians predominantly reported that they always check for these elements or when the patient needs it. The few that did not perform these care components did not rank these elements as the highest priority, indicating no discordance between priorities and care practices. 12.3% of OB/GYNs reported that they did not assess patients for *breast health/feeding* and among midwives 27.8% reported that they did not perform *family planning counseling* (Supplementary 2).

**Fig. 2:**
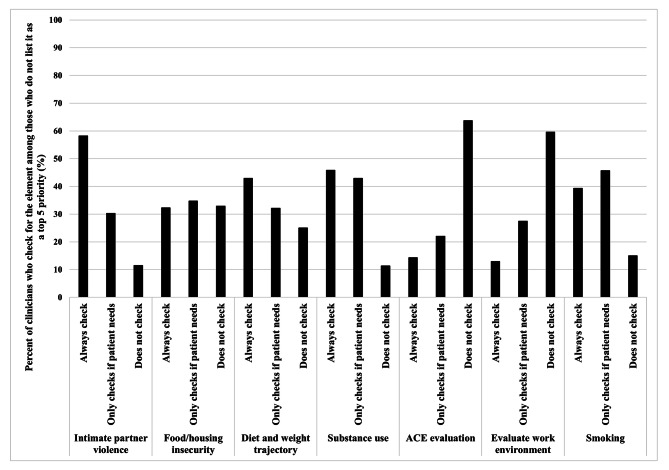
Clinician care practices among priorities rated as “top priorities” by ≤ 5% of all clinicians* *only the respondents who did not list the components in their top 5 priorities (≥ 95% of respondents) are shown in the figure

When we focused the analysis on the care components that only ≤ 5% of all clinicians ranked as highest priority, we found that among those clinicians who do not rank these components highly, there was a strong tendency to not complete clinical assessment of these components (range 11–64%) or to assess only when the patient needs it (Fig. 2). Among those clinicians who did not complete the assessment, the majority reported that they did not check for those components (ranging from 60% for intimate partner violence to 89.5% for substance use); few mentioned that they delegated or referred elsewhere (results not shown).

We additionally compared the highest priorities identified by OB/GYNs and midwives with those ranked according to practice characteristics, namely practice setting, practice location, volume of patients with Medi-Cal insurance seen and timing of the first postpartum visit. We found several care components highly prioritized by both OB/GYNs and midwives that were similarly prioritized across these practice characteristics (Table 3). Specifically, *depression and anxiety*,* breast health/feeding and vaginal birth complications* consistently ranked in the highest tier. *C-section birth complications and pregnancy-related complications* which were ranked highly by OB/GYNs (but not by midwives consistent with their scope of practice) were also ranked highly by clinicians employed in large cities or suburban locations and by clinicians who see patients later than 2 weeks or depending on patient needs. These practice characteristics correspond closely to those of OB/GYNs as shown in Table 1.

**Table 3:** Top 5 priorities by clinician type and practice characteristics

Clinician Priority	Clinician Type		Practice Setting					Practice Location			Timing of First Postpartum Visit				% Patients on Medi-Cal	
	OB/GYNs	Midwives	University/Academic hospital	Community hospital-health center-birthing center	Staff Model HMO	Private Practice	Multi-Affiliated/Other	Urban/ large city	Suburb, near large city	Small city or town	Within 2 weeks	Within 3–5 weeks	Within 6–12 weeks	Depends on patient’s condition	≤ 50%	> 50%
Depression and anxiety	✓	✓	✓	✓	✓	✓	✓	✓	✓	✓	✓	✓	✓	✓	✓	✓
Breast health, feeding issues	✓	✓	✓	✓	✓	✓	✓	✓	✓	✓	✓	✓	✓	✓	✓	✓
Vaginal birth complications	✓	✓	✓	✓	✓	✓	✓	✓	✓		✓	✓	✓	✓	✓	✓
C-section birth complications	✓		✓	✓	✓		✓	✓	✓			✓	✓	✓	✓	✓
Pregnancy-related complications	✓		✓	✓	✓			✓	✓			✓	✓	✓	✓	✓
Family planning counseling	✓	✓	✓		✓			✓	✓			✓	✓	✓		✓
Social and emotional support		✓		✓			✓		✓	✓	✓		✓	✓	✓	✓
Physical recovery after labor		✓		✓		✓			✓	✓	✓				✓	
Maternal sleep		✓				✓										

In contrast, *physical recovery after labor* was more likely to be highly prioritized by clinicians who practice in community settings and in private practice, in suburbs or small towns, serving ≤ 50% of Medi-Cal patients and who see patients in the first two weeks or depending on patient’s needs. Most of these practice characteristics are associated with midwifery practice. Similarly, *social/emotional support* was highly prioritized by clinicians practicing in community settings and suburbs or small towns and *maternal sleep* was highly prioritized by clinicians in private practice, all aligned with midwifery practice. Notably, we found no differences in the priorities of clinicians in practices with a low or high volume of patients with Medi-Cal insurance, except for the emphasis on *physical recovery after labor* among low volume Medi-Cal practices versus an emphasis placed on *family planning counseling* among clinicians serving > 50% with patients with Medi-Cal insurance.

## Discussion

We conducted an online survey of current clinician priorities and practices in California and found that clinicians rarely performed *all* postpartum care components recommended by ACOG. Nonetheless, what the clinicians did prioritize highly, they were likely to perform. OB/GYNs and midwives had similar priorities for postpartum care, except for those clinical components that fell outside midwives’ scope of practice and a few other components that only midwives valued highly. This survey took place in the context of scarce data on the optimal timing, frequency, and content of postpartum visits, along with the inconsistent recommendations for standards of care for the fourth trimester. This care inconsistency affects a lot of patients. About 1 in 10 births nationwide occur in California. 98% of these births occur in a hospital; approximately 86% are attended by physicians and 14% by midwives [5]. In 2018–2020 surveys of California mothers, 89% reported that they had a postpartum medical visit [5].

We found that d*epression and anxiety*,* breast health/breast feeding issues*,* vaginal birth complications and family planning counseling* stand out as consistently highly ranked components for the first outpatient postpartum visit, ranked highly across clinician types and practice characteristics. *Depression screening*,* family planning counseling* and *birth-related complications* were also found to be highly prioritized in a previous national survey of clinicians conducted in 2018 [11]. The data collected for that survey overlapped with the publication of the ACOG guidelines and were completed prior to NCQA setting depression screening and follow-up care and family planning counseling as a national standard for postpartum care [3, 4, 10].

Other top- ranked components are prioritized somewhat differently depending on clinician type. *C-section birth complications and pregnancy-related complications* were ranked highly by OB/GYNs, but not by midwives because these elements are outside the scope of practice for midwives. In contrast, *social and emotional support*,* physical recovery after labor and maternal sleep* were more highly ranked by midwives and are consistent with the relationship-centered model of midwifery care [14]. We also found that the high-ranking components observed among OB/GYNs and midwives are closely associated with the rankings observed according to the type of practice settings, practice locations and timing of initial postpartum care offered. It is plausible that these observed differences are associated with the differences in training, education, or specialization of different clinical elements. Alternatively, observed differences associated with patient management styles and emphases may be due to the various care settings in which the clinicians practice. It is also possible that different types of clinicians are influenced by their patients’ expectations of care. The extent to which clinician priorities align with their perceptions of patient priorities requires further investigation.

Clinicians are highly invested in the care practices that they identify as most important. The majority reported that these components are checked always or when the patient needs it, even if they themselves did not rank them as their top five priorities. Early identification and treatment of these conditions may help to prevent death and illness. However, there is limited evidence regarding the impact of clinical interventions on postpartum outcomes, an area warranting further study [15, 16].

In contrast, care components that very few clinicians consider a top priority more often go unchecked by them or performed only if the patient needs it. This seems to be the case for diet and weight trajectory and many care components that are social drivers of health, including screening and counseling for intimate partner violence, food and housing insecurity, substance use and smoking, ACEs, and assessment of the work environment, including maternity leave. It is interesting to note that despite midwives spending a median of 60 min with their patients at the first visit compared to 20 min spent by OB/GYNs, midwives are not more likely to prioritize the components of social drivers of health at the first visit. This would indicate that the length of visit is not the main barrier to prioritizing social drivers of health. Perhaps many of these recommended care components are prioritized and performed in subsequent postpartum care visits. This may be more probable among midwives than OB/GYNs, since midwives are more likely to provide more than one visit to their low-risk patients; midwives also tend to adhere to a relationship-centered model of care which may prompt them to check for these components in future contacts [13].

Alternatively, perhaps the clinicians are not performing these care components in the postpartum due to multiple competing demands and few clinic-based or community resources or because clinicians consider that patients may not be able to absorb multiple messages. Aside from assessing anxiety and depression - two prevalent conditions in the postpartum that are often reimbursed by health plans- other mental health and social issues might be considered too time intensive to approach, causing providers to avoid raising them if possible [2]. Future studies need to explore these types of barriers.

Since social drivers influence disease risk and susceptibility, including disparities in obstetric outcomes and wellbeing after birth, the related care components should be addressed to achieve comprehensive care [17, 18]. Considering that clinicians overall report that one in three of their low-risk patients are seen for a single visit, and that among OB/GYNs this prevalence may be as high as 46%, these elements are likely to be underperformed.

Finally, we examined the extent to which clinicians serving a high percent of Medi-Cal patients prioritize similar care components as those serving a low volume of patients with Medi-Cal insurance, and found few differences, suggesting comparable care. Medi-Cal is an important funder of postpartum care, insuring 40% of the deliveries in California [5]. Evidence shows that patients with Medi-Cal insurance tend to require more support with social drivers of care, suggesting that this is an area for improvement to achieve equitable and positive health outcomes [18]. In the future we plan to explore more fully how clinicians decide on the tradeoffs they make.

### Strengths and limitations

Our near-time study had several strengths, including the high response rate and diversity in racial, age and practice characteristics of study participants. We examined multiple components of postpartum care and went beyond binary measures of whether clinicians do or do not provide care by asking them if they provide care always or when the patient needs it. By asking respondents to rank the top five components from a list, we could easily identify the elements that rise to the top. This helped to mitigate a common social desirability bias that occurs when respondents answer questions in ways that are viewed favorably by others, concealing their true experiences [19].

Our study also had some limitations. We drew a convenience sample that may represent motivated clinicians who have strong opinions, or strive for quality improvement in the postpartum, and we cannot generalize our findings to all practicing clinicians in California. Furthermore, our sample was underpowered to detect small differences in outcomes and we did not capture all elements of care. We asked about general postpartum priorities and practices and did not ask about familiarity with multiple guidelines or their applications under different clinical circumstances. Finally, we only focused on the content of the first postpartum visit and did not capture the care received in follow-up visits.

### Implications for clinical practice

Increasingly, there is an understanding of the importance of fourth trimester care to support growing families and address important health issues before they become intergenerational. It should come as no surprise to clinicians that a single postpartum visit –which represents a common model of care in California- is not enough to cover all the identified important components of comprehensive postpartum care. Clinicians therefore make tradeoffs based on their priorities and may choose specific components to focus on or may even try to add additional visits that are not fully reimbursed to cover other components.

Individually, we understand that each care component is an important contributor to good health, but more data are needed to understand how important it is to perform all the care elements. Postpartum care offers a unique opportunity to provide important screening for substance abuse, intimate partner violence and to address obesity and other medical comorbidities; however, clinicians face barriers to completing all the necessary items in one visit. This begs the question of the nature of the postpartum visit and whether it is a preventive visit, a problem-driven acute care visit, or a procedural follow-up visit and whether any of those models truly address the care needed at this vulnerable time.

At a time when CAL AIM (California Advancing and Innovating Medi-CAL) pays health plans to contract with community-based and social services providers to address social drivers to achieve care equity, it is revealing to know that many of these social drivers are not prioritized and therefore not assessed by obstetric clinicians in the first—and for many patients the single—postpartum visit.

Our results suggest that a collaborative approach, bringing together different types of clinicians with different priorities, might mitigate gaps in care. Midwives and physicians who collaborate with social workers, doulas, nutritionists, mental health, and community health workers might better cover the spectrum of post-partum care recommendations. Further research is needed to understand the cost benefit of a collaborative post-partum care model. We find it reassuring that the payor source does not seem to be a strong factor in setting priorities, as Medi-Cal is such a prevalent source of pregnancy and postpartum funding. This gives us hope that with the expanded Medicaid and Medical coverage, improved models of postpartum care may be considered.

In conclusion, we found that, despite professional societies’ recommendations, clinicians are unable to complete all the items recognized as essential to postpartum care with the time and resources available to them during a single postpartum visit. Further research that examines the extent to which other components are completed at subsequent visits or by different health care providers is warranted. With extended Medicaid insurance for postpartum care up to 12 months in California and other states, there is a need to understand what care is being provided, what gaps remain and whether collaborative models of care may provide comprehensive and quality care. We performed a near-time survey to understand current obstetric clinician priorities and what care was provided at the first postpartum visit in California. Our findings highlight that priorities for midwives and OB/GYNs track their scope of practice, and clinicians do actualize the care they prioritize highly. These findings can serve as a springboard to understanding postpartum care practices and may inform future studies to identify remaining gaps in postpartum care and suggest improved models of care.

### Electronic supplementary material

Below is the link to the electronic supplementary material.

Supplementary Material 1

Supplementary Material 2

## Data Availability

The data analyzed during the current study is mostly included in this article and its supplementary file. Data are available from the corresponding author upon reasonable request.

## Abbreviations

ACOG: American College of Obstetricians and Gynecologists
OB/GYN: Obstetrician and Gynecologist
Medi: Cal–Medicaid insurance coverage in California
NCQA: National Committee for Quality Assurance
CMQCC: California Maternal Health Quality Control Collaborative
MOD: March of Dimes
CDPH: California Department of Public Health
ACEs: Adverse childhood experiences
CAL AIM: California Advancing and Innovating Medi–CAL
N.S.: Non–significant result at an alpha level of 0.05
