# Supplementary material for: Clinician care priorities and practices in the fourth trimester: perspective from a California survey
Source: BMC Pregnancy Childbirth. 2024 Jul 25;24:502. doi: 10.1186/s12884-024-06705-7 (PMC11274747; doi:10.1186/s12884-024-06705-7)
Supplement: Supplementary file 1 — Supplementary Material 1 [file 12884_2024_6705_MOESM1_ESM.pdf]

Block 1
You are invited as a provider of obstetric care to participate in a research study on ways to build a better system of care during the postpartum in California. The survey will ask you questions about your current practices, priorities, patient preferences, gaps and opportunities to improve delivery of care in the postpartum.
Block 3
Let's see if you qualify, by answering the following 3 questions:

Do you personally treat patients in the postpartum period?

- ○ Yes
- ○ No

What type of maternity provider are you (select one of the following)

- ○ Obstetrician and gynecologist (OB-GYN)
- ○ Family practitioner
- ○ Maternal-fetal Medicine specialist (MFM)
- ○ Medical resident
- ○ Clinical fellow
- ○ Certified Nurse Midwife
- ○ Physician Assistant
- ○ Other ______

Do you currently practice in California?

- ○ Yes
- ○ No

Block 4

Success, you qualify! Can you please give us your e-mail address to enter it to the $100 gift drawing? (All emails will be deleted once the drawing is completed).

Email Address ______

I am sorry but you do not meet the inclusion criteria for this study. Perhaps you could refer us to some of your colleagues. By providing us with at least 2 emails of potential participants we will put you in a special card drawing of $50.

Email Address #1 ______
Email Address #2 ______
Email Address #3 (Optional) ______

Block 8

Please review the consent form below. If you agree to participate in this study, check the box below.

Consent survey form.docx

- ○ Yes, I agree to participate in this Study
- ○ No, I do not want to participate in this Study

Block 2
We will begin with some questions about your current clinical practices.
Current Practices

In your practice, who provides the most primary postpartum care:

- ○ An OB/GYN

- ○ Another physician non OB/GYN
- ○ Nurse-midwife
- ○ Nurse practitioner
- ○ Physician assistant
- ○ Other (specify): ______

Tell us which of these categories best describes how you spend your time with your patients:

- ○ I see my patients primarily for the birth/delivery.
- ○ I see my patients throughout prenatal, intrapartum (labor/delivery) and during the postpartum.
- ○ I see my patients routinely throughout prenatal and then postpartum.
- ○ I see them primarily in the post-partum.
- ○ Other (specify): ______

In what timeframe do you typically see patients for their first postpartum visit?

- ○ Within 1 week
- ○ Within 2 weeks
- ○ Within 3 weeks - 5 weeks
- ○ 6 weeks - 12 weeks
- ○ Depends on patient's clinical condition
- ○ Other (specify): ______

On average, how many minutes do you spend with a patient at their postpartum visit?

______

How many follow-up visits after the first visit do you typically provide to your non-high-risk patients during the fourth trimester (ie. First 12 weeks postpartum)

______

About how many follow-up visits after the first visit do you typically provide to your high-risk patients during the fourth trimester (ie. 12 weeks postpartum)

______

Are these visits typically in person, via telehealth or phone or a hybrid of in person and telehealth/phone

- ○ In person
- ○ Via telehealth or phone
- ○ Hybrid

What type of "model of care" is used at your facility during the postpartum period? "Model of care" is described as the way health services are delivered. Is it mainly…

- ○ Individual in-person appointments only
- ○ Individual-in person appointment with home visiting
- ○ Group postpartum/well-child care (i.e. Centering parenting)
- ○ Dyadic maternity/pediatric visits
- ○ Other (Specify): ______

In your opinion, which of the following times for the first postpartum visit would provide the most effective time to begin postpartum care?

- ○ A visit less than 1 week postpartum
- ○ A visit 1-2 weeks postpartum

- ○ A visit 3 to 4 weeks postpartum
- ○ A visit 5-6 weeks postpartum
- ○ A visit later than 6 weeks postpartum
- ○ Postpartum visits only for those with specific medical concerns
- ○ Other(Specify):

In your opinion, what is the optimal time to begin speaking to your patient/clients about the postpartum period (i.e. postpartum recovery, infant feeding, etc.)?

- ○ 1-3 months pregnant
- ○ 4-6 months pregnant
- ○ 7-9 months pregnant
- ○ In the postpartum ward (right after birth)

In your opinion, what are the benefits of extending postpartum care services from 60 days to 12 months?

What information do you routinely get yourself or from your team about your socially vulnerable patients in the postpartum?

What information do you routinely get yourself or from your team about your socially vulnerable patients in the postpartum?

	Get	Don't get but need it to act upon	Don't get or don't want it
**Housing instability and utility help needs**	○ Housing instability and utility help needs Get	○ Housing instability and utility help needs Don't get but need it to act upon	○ Housing instability and utility help needs Don't get or don't want it
**Food insecurity**	○ Food insecurity Get	○ Food insecurity Don't get but need it to act upon	○ Food insecurity Don't get or don't want it
**Transportation barriers to care**	○ Transportation barriers to care Get	○ Transportation barriers to care Don't get but need it to act upon	○ Transportation barriers to care Don't get or don't want it
**Safety and violence risk assessment**	○ Safety and violence risk assessment Get	○ Safety and violence risk assessment Don't get but need it to act upon	○ Safety and violence risk assessment Don't get or don't want it
**Financial strain**	○ Financial strain Get	○ Financial strain Don't get but need it to act upon	○ Financial strain Don't get or don't want it
**Employment conditions, including paid family leave plans**	○ Employment conditions, including paid family leave plans Get	○ Employment conditions, including paid family leave plans Don't get but need it to act upon	○ Employment conditions, including paid family leave plans Don't get or don't want it
**Family and community support**	○ Family and community support Get	○ Family and community support Don't get but need it to act upon	○ Family and community support Don't get or don't want it
**Substance use**	○ Substance use Get	○ Substance use Don't get but need it to act upon	○ Substance use Don't get or don't want it
**Mental health**	○ Mental health Get	○ Mental health Don't get but need it to act upon	○ Mental health Don't get or don't want it
**Disabilities**	○ Disabilities Get	○ Disabilities Don't get but need it to act upon	○ Disabilities Don't get or don't want it
**Other (specify):**	○ Other (specify): Get	○ Other (specify): Don't get but need it to act upon	○ Other (specify): Don't get or don't want it

Do you check for the following elements at the first postpartum visit?

Do you check for the following elements at the **first** postpartum visit?

**Clinical Elements**	Yes, always	Yes, but only if the patient needs it	No, but I delegate/refer	No, I do not
**C-section birth complications (when c-section births do occur)**	○ C-section birth complications (when c-section births do occur) Yes, always	○ C-section birth complications (when c-section births do occur) Yes, but only if the patient needs it	○ C-section birth complications (when c-section births do occur) No, but I delegate/refer	○ C-section birth complications (when c-section births do occur) No, I do not
**Vaginal birth complications (when vaginal births do occur)**	○ Vaginal birth complications (when vaginal births do occur) Yes, always	○ Vaginal birth complications (when vaginal births do occur) Yes, but only if the patient needs it	○ Vaginal birth complications (when vaginal births do occur) No, but I delegate/refer	○ Vaginal birth complications (when vaginal births do occur) No, I do not
**Pregnancy-related complications**	○ Pregnancy-related complications Yes, always	○ Pregnancy-related complications Yes, but only	○ Pregnancy-related complications No, but I	○ Pregnancy-related complications No, I do not

	Yes, always	Yes, but only if the patient needs it	No, but I delegate/refer	No, I do not
		if the patient needs it	delegate/refer	
**Recovery after labor**	○ Recovery after labor Yes, always	○ Recovery after labor Yes, but only if the patient needs it	○ Recovery after labor No, but I delegate/refer	○ Recovery after labor No, I do not
**Physical/pelvic exam**	○ Physical/pelvic exam Yes, always	○ Physical/pelvic exam Yes, but only if the patient needs it	○ Physical/pelvic exam No, but I delegate/refer	○ Physical/pelvic exam No, I do not
**Remote blood pressure monitoring**	○ Remote blood pressure monitoring Yes, always	○ Remote blood pressure monitoring Yes, but only if the patient needs it	○ Remote blood pressure monitoring No, but I delegate/refer	○ Remote blood pressure monitoring No, I do not
**Chronic health conditions**	○ Chronic health conditions Yes, always	○ Chronic health conditions Yes, but only if the patient needs it	○ Chronic health conditions No, but I delegate/refer	○ Chronic health conditions No, I do not
**Behavioral**				
**Depression and anxiety**	○ Depression and anxiety Yes, always	○ Depression and anxiety Yes, but only if the patient needs it	○ Depression and anxiety No, but I delegate/refer	○ Depression and anxiety No, I do not
**Substance use**	○ Substance use Yes, always	○ Substance use Yes, but only if the patient needs it	○ Substance use No, but I delegate/refer	○ Substance use No, I do not
**Smoking**	○ Smoking Yes, always	○ Smoking Yes, but only if the patient needs it	○ Smoking No, but I delegate/refer	○ Smoking No, I do not
**Maternal sleep**	○ Maternal sleep Yes, always	○ Maternal sleep Yes, but only if the patient needs it	○ Maternal sleep No, but I delegate/refer	○ Maternal sleep No, I do not
**Diet and weight trajectory**	○ Diet and weight trajectory Yes, always	○ Diet and weight trajectory Yes, but only if the patient needs it	○ Diet and weight trajectory No, but I delegate/refer	○ Diet and weight trajectory No, I do not
**Exercise and nutrition**	○ Exercise and nutrition Yes, always	○ Exercise and nutrition Yes, but only if the patient needs it	○ Exercise and nutrition No, but I delegate/refer	○ Exercise and nutrition No, I do not
**Family Planning**				
**Family planning counsel**	○ Family planning counsel Yes, always	○ Family planning counsel Yes, but only if the patient needs it	○ Family planning counsel No, but I delegate/refer	○ Family planning counsel No, I do not
**Contraceptive provision**	○ Contraceptive provision Yes, always	○ Contraceptive provision Yes, but only if the patient needs it	○ Contraceptive provision No, but I delegate/refer	○ Contraceptive provision No, I do not
**Resuming sexual activity**	○ Resuming sexual activity Yes, always	○ Resuming sexual activity Yes, but only if the patient needs it	○ Resuming sexual activity No, but I delegate/refer	○ Resuming sexual activity No, I do not
**Infant Health**				
**Breast health, breastfeeding and other infant feeding issues**	○ Breast health, breastfeeding and other infant feeding issues Yes, always	○ Breast health, breastfeeding and other infant feeding issues Yes, but only if the patient needs it	○ Breast health, breastfeeding and other infant feeding issues No, but I delegate/refer	○ Breast health, breastfeeding and other infant feeding issues No, I do not
**Infant safe sleep**	○ Infant safe sleep Yes, always	○ Infant safe sleep Yes, but only if the patient needs it	○ Infant safe sleep No, but I delegate/refer	○ Infant safe sleep No, I do not
**Infant bonding**	○ Infant bonding Yes, always	○ Infant bonding Yes, but only if the patient needs it	○ Infant bonding No, but I delegate/refer	○ Infant bonding No, I do not
**Social**				
**Social and emotional support**	○ Social and emotional support Yes, always	○ Social and emotional support Yes, but only if the patient needs it	○ Social and emotional support No, but I delegate/refer	○ Social and emotional support No, I do not
**Intimate partner violence**	○ Intimate partner violence Yes, always	○ Intimate partner violence Yes, but only if the patient needs it	○ Intimate partner violence No, but I delegate/refer	○ Intimate partner violence No, I do not
**Safe work environment**	○ Safe work environment Yes, always	○ Safe work environment Yes, but only if the patient needs it	○ Safe work environment No, but I delegate/refer	○ Safe work environment No, I do not
**Evaluate work environment**	○ Evaluate work environment Yes, always	○ Evaluate work environment Yes, but only if the patient needs it	○ Evaluate work environment No, but I delegate/refer	○ Evaluate work environment No, I do not
**ACE evaluation**	○ ACE evaluation Yes, always	○ ACE evaluation Yes, but only if the patient needs it	○ ACE evaluation No, but I delegate/refer	○ ACE evaluation No, I do not
**Food and/or housing insecurity**	○ Food and/or housing insecurity Yes, always	○ Food and/or housing insecurity Yes, but only if the patient needs it	○ Food and/or housing insecurity No, but I delegate/refer	○ Food and/or housing insecurity No, I do not
**Future Care**				
**Review person's birth experience and prepare for future pregnancies**	○ Review person's birth experience and prepare for future pregnancies Yes, always	○ Review person's birth experience and prepare for future pregnancies Yes, but only if the patient needs it	○ Review person's birth experience and prepare for future pregnancies No, but I delegate/refer	○ Review person's birth experience and prepare for future pregnancies No, I do not
**Development/implementation of a postpartum care plan that details patient's need for support, recovery, bonding, family planning and more)**	○ Development/implementation of a postpartum care plan that details patient's need for support, recovery, bonding,	○ Development/implementation of a postpartum care plan that details patient's need for support, recovery, bonding,	○ Development/implementation of a postpartum care plan that details patient's need for support, recovery, bonding,	○ Development/implementation of a postpartum care plan that details patient's need for support, recovery, bonding,

	Yes, always	Yes, but only if the patient needs it	No, but I delegate/refer	No, I do not
	family planning and more) Yes, always	family planning and more) Yes, but only if the patient needs it	family planning and more) No, but I delegate/refer	family planning and more) No, I do not
**Transitioning to primary care**	○ Transitioning to primary care Yes, always	○ Transitioning to primary care Yes, but only if the patient needs it	○ Transitioning to primary care No, but I delegate/refer	○ Transitioning to primary care No, I do not

What are the top 5 postpartum topics that you prioritize reviewing with your patient during the first postpartum visit? (Click on 5 items from the entire list below)

- **Clinical Elements**
  - ☐ C-section birth complications (when c-section births do occur)
  - ☐ Vaginal birth complications (when vaginal births do occur)
  - ☐ Pregnancy-related complications
  - ☐ Recovery after labor
  - ☐ Chronic health conditions
- **Behavioral**
  - ☐ Depression and anxiety
  - ☐ Substance use
  - ☐ Smoking
  - ☐ Maternal sleep
  - ☐ Diet and weight trajectory
  - ☐ Exercise and nutrition
- **Family Planning**
  - ☐ Family planning counsel
  - ☐ Contraceptive provision
  - ☐ Resuming sexual activity
- **Infant Health**
  - ☐ Breast health, breastfeeding and other infant feeding issues
  - ☐ Infant safe sleep
  - ☐ Infant bonding
- **Social**
  - ☐ Social and emotional support
  - ☐ Intimate partner violence
  - ☐ Safe work environment
  - ☐ Evaluate work environment
  - ☐ ACE evaluation
  - ☐ Food and/or housing insecurity
- **Future Care**
  - ☐ Review person's birth experience and prepare for future pregnancies
  - ☐ Development/implementation of a postpartum care plan that details patient's need for support, recovery, bonding, family planning and more)
  - ☐ Transitioning to primary care

In your experience, please indicate the extent to which your patients prioritize the following topics in their first postpartum visit:

In your experience, please indicate the extent to which your **patients** prioritize the following topics in their first postpartum visit:

	Not at all	Slightly	Moderately	Very	Extremely
**Clinical elements**	○ Clinical elements Not at all	○ Clinical elements Slightly	○ Clinical elements Moderately	○ Clinical elements Very	○ Clinical elements Extremely
**Behavioral**	○ Behavioral Not at all	○ Behavioral Slightly	○ Behavioral Moderately	○ Behavioral Very	○ Behavioral Extremely
**Family planning**	○ Family planning Not at all	○ Family planning Slightly	○ Family planning Moderately	○ Family planning Very	○ Family planning Extremely
**Infant health**	○ Infant health Not at all	○ Infant health Slightly	○ Infant health Moderately	○ Infant health Very	○ Infant health Extremely
**Employment Issues**	○ Employment Issues Not at all	○ Employment Issues Slightly	○ Employment Issues Moderately	○ Employment Issues Very	○ Employment Issues Extremely
**Social needs**	○ Social needs Not at all	○ Social needs Slightly	○ Social needs Moderately	○ Social needs Very	○ Social needs Extremely
**Future care**	○ Future care Not at all	○ Future care Slightly	○ Future care Moderately	○ Future care Very	○ Future care Extremely

How satisfied are you with the postpartum care that you are able to provide?

- ○ Not at all

- ○ Slightly
- ○ Moderately
- ○ Very
- ○ Extremely

Now we would like to find out about your experiences with barriers to care and care coordination.
Barriers to Care and Care Coordination

How often do you encounter the following barriers when providing postpartum care?

How often do you encounter the following barriers when providing postpartum care?

	Never	Rarely	Sometimes	Often	Always
**Organizational/Systemic barriers (eg.poor care coordination, limited access to referrals & services, limited clinic hours, excessive paperwork)**	○ Organizational/Systemic barriers (eg.poor care coordination, limited access to referrals & services, limited clinic hours, excessive paperwork) Never	○ Organizational/Systemic barriers (eg.poor care coordination, limited access to referrals & services, limited clinic hours, excessive paperwork) Rarely	○ Organizational/Systemic barriers (eg.poor care coordination, limited access to referrals & services, limited clinic hours, excessive paperwork) Sometimes	○ Organizational/Systemic barriers (eg.poor care coordination, limited access to referrals & services, limited clinic hours, excessive paperwork) Often	○ Organizational/Systemic barriers (eg.poor care coordination, limited access to referrals & services, limited clinic hours, excessive paperwork) Always
**Financial barriers (eg. insufficient or slow reimbursement, insurance disruptions)**	○ Financial barriers (eg. insufficient or slow reimbursement, insurance disruptions) Never	○ Financial barriers (eg. insufficient or slow reimbursement, insurance disruptions) Rarely	○ Financial barriers (eg. insufficient or slow reimbursement, insurance disruptions) Sometimes	○ Financial barriers (eg. insufficient or slow reimbursement, insurance disruptions) Often	○ Financial barriers (eg. insufficient or slow reimbursement, insurance disruptions) Always
**Patient constraints (eg. limited time, lack of knowledge, language differences, distrust)**	○ Patient constraints (eg. limited time, lack of knowledge, language differences, distrust) Never	○ Patient constraints (eg. limited time, lack of knowledge, language differences, distrust) Rarely	○ Patient constraints (eg. limited time, lack of knowledge, language differences, distrust) Sometimes	○ Patient constraints (eg. limited time, lack of knowledge, language differences, distrust) Often	○ Patient constraints (eg. limited time, lack of knowledge, language differences, distrust) Always
**Other (Specify)** ____	○ Other (Specify) Never	○ Other (Specify) Rarely	○ Other (Specify) Sometimes	○ Other (Specify) Often	○ Other (Specify) Always

Who do you currently collaborate (i.e. work regularly with, send/receive referrals, and/or coordinate patient care) with during the postpartum period of care?

Who do you currently collaborate (i.e. work regularly with, send/receive referrals, and/or coordinate patient care) with during the postpartum period of care?

	Yes	No, but Interested in a collaboration	Not interested in a collaboration
**Doulas**	○ Doulas Yes	○ Doulas No, but Interested in a collaboration	○ Doulas Not interested in a collaboration
**Lactation Consultants**	○ Lactation Consultants Yes	○ Lactation Consultants No, but Interested in a collaboration	○ Lactation Consultants Not interested in a collaboration
**Nutritionists/dieticians**	○ Nutritionists/dieticians Yes	○ Nutritionists/dieticians No, but Interested in a collaboration	○ Nutritionists/dieticians Not interested in a collaboration
**Psychiatrists**	○ Psychiatrists Yes	○ Psychiatrists No, but Interested in a collaboration	○ Psychiatrists Not interested in a collaboration
**Psychologists/ Therapists**	○ Psychologists/ Therapists Yes	○ Psychologists/ Therapists No, but Interested in a collaboration	○ Psychologists/ Therapists Not interested in a collaboration
**Social Workers**	○ Social Workers Yes	○ Social Workers No, but Interested in a collaboration	○ Social Workers Not interested in a collaboration
**Other (Specify):** ____	○ Other (Specify): Yes	○ Other (Specify): No, but Interested in a collaboration	○ Other (Specify): Not interested in a collaboration

At your organization, are postpartum patients linked to Primary Care Physicians (PCPs)?

- ○ My patients are typically connected to in-network PCPs
- ○ My patients are typically connected to in-or–out of network PCPs
- ○ We do not follow up with patients about this
- ○ Unsure or don't know

Block 4

How often are you able to successfully refer a patient to the following services?

How often are you able to successfully refer a patient to the following services?

	Never	Rarely	Sometimes	Often	Always
**Mental health**	○ Mental health Never	○ Mental health Rarely	○ Mental health Sometimes	○ Mental health Often	○ Mental health Always
**Lactation**	○ Lactation Never	○ Lactation Rarely	○ Lactation Sometimes	○ Lactation Often	○ Lactation Always

	Never	Rarely	Sometimes	Often	Always
**Pelvic floor or urology**	○ Pelvic floor or urology Never	○ Pelvic floor or urology Rarely	○ Pelvic floor or urology Sometimes	○ Pelvic floor or urology Often	○ Pelvic floor or urology Always
**Chronic care specialists (eg. diabetes, hypertension)**	○ Chronic care specialists (eg. diabetes, hypertension) Never	○ Chronic care specialists (eg. diabetes, hypertension) Rarely	○ Chronic care specialists (eg. diabetes, hypertension) Sometimes	○ Chronic care specialists (eg. diabetes, hypertension) Often	○ Chronic care specialists (eg. diabetes, hypertension) Always
**Social services**	○ Social services Never	○ Social services Rarely	○ Social services Sometimes	○ Social services Often	○ Social services Always
**Substance abuse clinics**	○ Substance abuse clinics Never	○ Substance abuse clinics Rarely	○ Substance abuse clinics Sometimes	○ Substance abuse clinics Often	○ Substance abuse clinics Always

Block 5
Reimbursement

Do you generally get reimbursed for postpartum care through a:

- ○ Fee for service (pay per visit)
- ○ Global maternity fee (fixed fee per patient for prenatal, delivery & postpartum)
- ○ Fixed salary
- ○ Fixed salary plus incentives for value added care
- ○ Other: Specify

Do you feel your pay for postpartum care is underpaid, overpaid or just about right?

- ○ Underpaid
- ○ Overpaid
- ○ Just about right

Block 6
Almost there!!
Demographics

What is your age? (in years)

What is your gender?

- ○ Woman
- ○ Man
- ○ Transgender
- ○ Non-binary/non-conforming
- ○ Other:
- ○ Prefer not to respond

What is your race:

- ○ Black
- ○ White
- ○ Asian
- ○ Native American
- ○ Native Hawaiian/Pacific Islander
- ○ Other(specify):

Are you of Hispanic, Latino/a/x, or of Spanish origin?

- ○ Yes
- ○ No

Final questions, please complete!
Clinical Experience/Practice Setting

How many years have you been practicing with patients during the postpartum period (after completion of residence):

Which category best describes your practice setting (can choose more than one if practice is in multiple settings):

- ☐ University/Academic hospital
- ☐ Community hospital/FQHC
- ☐ Community health center or clinic
- ☐ Staff Model HMO (eg. Kaiser)
- ☐ Private practice
- ☐ Other non-affiliated:

In what location do you usually practice?

- ○ Urban, large city
- ○ Suburb, near large city
- ○ Small city or town
- ○ Rural

What percentage of your patients have Medi-Cal? (Provide best estimate)

- ○ Under 25%
- ○ 25-50%
- ○ 51-75%
- ○ Over 75%
- ○ Do not know

What percentage of your patients have CHIP? (Provide best estimate)

- ○ Under 25%
- ○ 25-50%
- ○ 51-75%
- ○ Over 75%
- ○ Do not know

Please identify the racial/ethnic makeup of your patient population by ascribing a % (Provide best estimate)

Please identify the racial/ethnic makeup of your patient population by ascribing a % (Provide best estimate)

	**Under 25%**	**25-50%**	**51-75%**	**Over 75%**
**African American or Black**	○ African American or Black Under 25%	○ African American or Black 25-50%	○ African American or Black 51-75%	○ African American or Black Over 75%
**American Indian or Alaska Native**	○ American Indian or Alaska Native Under 25%	○ American Indian or Alaska Native 25-50%	○ American Indian or Alaska Native 51-75%	○ American Indian or Alaska Native Over 75%
**White**	○ White Under 25%	○ White 25-50%	○ White 51-75%	○ White Over 75%
**Asian**	○ Asian Under 25%	○ Asian 25-50%	○ Asian 51-75%	○ Asian Over 75%
**Native Hawaiian and other Pacific islander**	○ Native Hawaiian and other Pacific islander Under 25%	○ Native Hawaiian and other Pacific islander 25-50%	○ Native Hawaiian and other Pacific islander 51-75%	○ Native Hawaiian and other Pacific islander Over 75%
**Hispanic, Latino/a/x**	○ Hispanic, Latino/a/x Under 25%	○ Hispanic, Latino/a/x 25-50%	○ Hispanic, Latino/a/x 51-75%	○ Hispanic, Latino/a/x Over 75%

Block 7

Thank you for your generous time to complete the survey. Your responses are very valuable and will help us advocate for an improved delivery system for postpartum care. If you have further comments please write them here:

Re-Contact

Would you like to be contacted again for additional information related to this postpartum research

- ◯ Yes
- ◯ No

Please provide the best email address and phone number to reach you in the near future:

Email
Number

Would like to receive a report on our findings when completed?

- ◯ Yes
- ◯ No

Would you like to join our Advisory Council?

- ◯ Yes
- ◯ No

Powered by Qualtrics
