# Supplementary material for: Clinician care priorities and practices in the fourth trimester: perspective from a California survey
Source: BMC Pregnancy Childbirth. 2024 Jul 25;24:502. doi: 10.1186/s12884-024-06705-7 (PMC11274747; doi:10.1186/s12884-024-06705-7)
Supplement: Supplementary file 2 — Supplementary Material 2 [file 12884_2024_6705_MOESM2_ESM.docx]

S-2. Clinician care practices associated with top priorities among all clinicians

|  |  | **All^†^** | | | **OB/GYNs** | | | **Midwives** | | |
| --- | --- | --- | --- | --- | --- | --- | --- | --- | --- | --- |
| **Clinician Priority** |  | **List as Top 5 (%)** | **Does not list as Top 5 (%)** | **p-value** | **List as Top 5 (%)** | **Does not list as Top 5 (%)** | **p-value** | **List as Top 5 (%)** | **Does not list as Top 5 (%)** | **p-value** |
| **Depression & anxiety** | **Always check** | 97.3 | 89.3 | N.S. | 97.7 | 100 | N.S. | 95.6 | 81.3 | N.S. |
|  | **Only checks if patient needs** | 2.7 | 10.7 |  | 2.3 | 0 |  | 4.4 | 18.8 |  |
|  | **Does not check** | 0 | 0 |  | 0 | 0 |  | 0 | 0 |  |
| **Breast** **health/feeding** | **Always check** | 96.3 | 76.9 | <0.0001 | 100 | 75.4 | <0.01 | 94.6 | 100 | N.S. |
|  | **Only checks if patient needs** | 3.7 | 12.3 |  | 0 | 12.3 |  | 5.4 | 0 |  |
|  | **Does not check** | 0 | 10.8 |  | 0 | 12.3 |  | 0 | 0 |  |
| **Vaginal birth complications** | **Always check** | 95.6 | 95.2 | N.S. | 96.6 | 94.7 | N.S. | 92 | 94.4 | N.S. |
|  | **Only checks if patient needs** | 4.4 | 4.8 |  | 3.4 | 5.3 |  | 8 | 5.6 |  |
|  | **Does not check** | 0 | 0 |  | 0 | 0 |  | 0 | 0 |  |
| **C-section birth complications** | **Always check** | 92 | 93.1 | N.S. | 90.5 | 97.1 | N.S. | 93.3 | 89.1 | N.S. |
|  | **Only checks if patient needs** | 8 | 6.9 |  | 9.5 | 2.9 |  | 6.7 | 10.9 |  |
|  | **Does not check** | 0 | 0 |  | 0 | 0 |  | 0 | 0 |  |
| **Pregnancy-related complications** | **Always check** | 92.5 | 91.5 | N.S. | 93.8 | 87.5 | N.S. | 80 | 92.2 | N.S. |
|  | **Only checks if patient needs** | 7.5 | 8.5 |  | 6.2 | 12.5 |  | 20 | 7.8 |  |
|  | **Does not check** | 0 | 0 |  | 0 | 0 |  | 0 | 0 |  |
| **Family planning counsel** | **Always check** | 94.8 | 82.5 | <0.01 | 95.7 | 98 | N.S. | 92 | 55.6 | <0.01 |
|  | **Only checks if patient needs** | 5.2 | 7.2 |  | 4.3 | 2 |  | 8 | 16.7 |  |
|  | **Does not check** | 0 | 10.3 |  | 0 | 0 |  | 0 | 27.8 |  |
| **Social & emotional support** | **Always check** | 93.2 | 73 | <0.01 | 93.8 | 64.1 | <0.01 | 94.1 | 88.9 | N.S. |
|  | **Only checks if patient needs** | 6.8 | 22 |  | 6.3 | 31.3 |  | 5.9 | 7.4 |  |
|  | **Does not check** | 0 | 5 |  | 0 | 4.7 |  | 0 | 3.7 |  |
| **Recovery after labor** | **Always check** | 97.1 | 84.2 | <0.05 | 95.7 | 84.7 | N.S. | 97.5 | 85 | N.S. |
|  | **Only checks if patient needs** | 2.9 | 12.9 |  | 4.3 | 13.9 |  | 2.5 | 10 |  |
|  | **Does not check** | 0 | 3 |  | 0 | 1.4 |  | 0 | 5 |  |

*N.S. indicates a non-significant result (p-value > 0.05)

^†^All clinicians includes OB/GYNs, Midwives, Family medicine doctors, and Nurse practitioners
